# State Dental Association

**Published:** 1898-06

**Authors:** 


### STATE DENTAL ASSOCIATION.

The following have been elected as officers of the Maryland State Dental Association for the ensuing year: President, Dr. M. Gist Sykes of Ellicott City; Dr. E. Cruzen, first vice-president; Dr. Geo. R. Carter, second vice-president; Dr. W. W. Dunbracce, corresponding secretary; Dr. Richard Grady, recording secretary; Dr. S. C. Pennington, treasurer. Executive committee, Drs. David Genese, L. W. Davis and H. S. Abendschein.

